# Determinants of Self-Care and Home-Based Management of Hypertension: An Integrative Review

**DOI:** 10.5334/gh.1190

**Published:** 2023-03-20

**Authors:** Kennedy Diema Konlan, Jinhee Shin

**Affiliations:** 1 Mo-Im Kim Nursing Research Institute, Yonsei University College of Nursing, 50-1, Yonsei-ro, Seodaemun-gu, Seoul 03722, Korea; 2 Department of Public Health Nursing, School of Nursing and Midwifery, University of Health and Allied Sciences, Ho, Ghana; 3 College of Nursing, Woosuk University, Jeollabuk-do, 55338, Korea

**Keywords:** home-based management, self-care, hypertension, treatment, adherence, care

## Abstract

**Introduction:**

Patients with hypertension should perform diverse self-care activities that incorporate medication adherence and lifestyle modification, such as no smoking or alcohol, weight reduction, a low-salt diet, increased physical activity, increased self-monitoring, and stress reduction, for effective management at home.

**Aim:**

This systematic review assessed and synthesized the factors that are associated with self-care and home-based management of hypertension.

**Methods:**

The search of the articles incorporated the population, intervention, comparison, and outcome (PICO) framework. The literature was searched in four databases (PubMed, the Cumulative Index to Nursing and Allied Health Literature [CINAHL], Embase, and Web of Science) until 2022. The articles retrieved and searched from the reference list (531) were transported to EndNote version 20, and duplicates (19) were identified and removed to produce 512 titles. Following the eventual title, abstracts, and full-text screening, 13 articles were appropriate for this study. The narrative and thematic data analysis were used to analyze and integrate the data.

**Results:**

The analysis showed five themes were associated with home-based self-care and blood pressure (BP) control among patients diagnosed with hypertension. These themes that emerged were (1) the prevalence of control of BP, (2) sociodemographic factors, (3) treatment-related factors, (4) knowledge of management, and (5) knowledge of the prevention of risk factors of hypertension. The demographic factors influencing home-based self-care for hypertension were gender, age, and socioeconomic status. In contrast, the treatment factors were duration of hypertension treatment, medication burden, and medication adherence. Other factors that influenced self-care were inadequate knowledge of BP management, follow-up care, and risk factors of hypertension.

**Conclusion:**

Hypertension self-care interventions must incorporate individual, societal, and cultural perspectives in increasing knowledge and improving home-based hypertension management. Therefore, well-designed clinical and community-dwelling interventions should integrate personal, social, and cultural perspectives to improve behavior in the home management of hypertension by increasing knowledge and self-efficacy.

## Introduction

Hypertension disease presents a challenge to patients, as they are expected to institute measures at home to ensure effective self-care and management practices. Patients with hypertension perform diverse activities that can be described as self-care activities for effective disease management [1]. The focus of hypertension self-care management must incorporate medication adherence and lifestyle modification (no smoking or alcohol, weight reduction, low-salt diet, and increased physical activity), increased self-monitoring of blood pressure (BP), and stress reduction [2]. However, self-care management in hypertensive patients must be lifelong, even though it is usually challenging and overwhelming because the patient lacks experience in self-management and the necessary knowledge, tools, and support [3]. Self-care is described as individual actions directed toward self or the environment to regulate individual functioning to improve health, reduce risk, and avoid related complications, as well as ensure general well-being [4,5]. It is important to note that self-care behavior among hypertension patients relates to BP control and prevents related complications [5]. Patient self-care and home-based management of hypertension positively affect clinical outcomes and reduce the occurrence of stroke and related cardiovascular disease [2,5]. Home-based measures are important in the patient’s likelihood of avoiding complications and effective improvement [2,4,6]. Self-care adherence is low among adults with hypertension [7] because patients are often unwilling to make recommended behavioral changes [8,9], especially in settings where lay health knowledge is averagely low.

Globally, the prevalence of hypertension has increased and shifted from developed to developing countries over the past 40 years [10]. Nearly three-quarters of hypertensive patients live in developing countries [11]. Awareness of high BP management is deficient in developing countries [12,13]. Many hypertensive patients are ignorant of their disease and treatment due to lack of health care access, distrust of Western medicine, and inadequate health literacy [12,14]. Although adherence to self-care and management is an important part of patient management to achieve hypertension treatment goals, self-care in the African population remains poor [15,16]. Therefore, it is very important to identify factors that influence hypertension self-care and management at home.

The self-care and home management of hypertension, like other chronic diseases, involve various behavioral changes that require optimal and effective medication adherence, self-efficacy, and prevention of complications [6,8,17]. Recent systematic reviews on self-care management of hypertension focused on the use of electronic-based technologies [18,19,20], the influence of self-care efficacy [6], and health promotion interventions for the prevention of hypertension [16,17,21]. However, to the best of our knowledge, no systematic review in recent times has specifically focused on the factors that influence home-based and self-care management of hypertension. This systematic review assessed and thematically synthesized the factors that are associated with self-care and home-based management of hypertension.

## Methods

### Study framework

The search of the articles incorporated the population, intervention, comparison, and outcome (PICO) framework. The population was patients diagnosed with hypertension and referred to home care or self-care management. There were no specific comparisons. The outcome was improved hypertension status or a reduction in BP. The Preferred Reporting Items for Systematic Reviews and Meta-Analyses (PRISMA) framework was incorporated in reporting the studies. Using the PRISMA guidelines and checklist allowed for reproducibility, transparency, and clarity in reporting the findings [22,23]. The phases of the entire study included identifying the research question, identifying relevant studies, quality appraisal, and selecting studies for inclusion.

### Identification of the research question

Home and self-care management of hypertension is an essential factor that can promote the health of people diagnosed with the disease, especially in the poorest parts of the world, like sub-Saharan Africa. Therefore, interventions must be tailored toward addressing the specific determinants of hypertension self-care management. The specific research question was, what are the determinants of self-care and home management of hypertension in low-resource settings in Africa?

### Identification of relevant studies

The literature search was done in four databases (PubMed, the Cumulative Index to Allied Health and Nursing Research [CINAHL], Embase, and Web of Science) up until 2022. In developing the search terms, the medical subject heading (MeSH) terms were used as the bases as well as free terms and wildcards incorporated with the appropriate Boolean operators. The search keywords were modified with the appropriate keywords to reflect the specific requirement of each database. The key terms used were first generated in PubMed and subsequently modified to sweet for each database. These primary keywords from PubMed using the PICO framework include population as “Hypertension”[MeSH] OR “High Blood Pressure” OR “Heart Disease”; Interventions as (“Self-Care”[MeSH] OR “Home Care Services”[MeSH] OR “Disease Management”[MeSH] OR “Therapeutics”[MeSH]); Comparison (Determinant* OR Predictor* OR Related OR Associated); and Outcome as “Medication Adherence” OR “Dietary Restriction” OR “Improved Physical Activity” OR “Increased Knowledge” OR “Improved Awareness.” These keywords were combined with the appropriate Boolean operators and MeSH terms where it was applicable.

### Selection of studies

After conducting the initial search, each title, abstract, and full text was screened based on predetermined inclusion criteria and the identified key terms. Also, the references of selected articles were also searched to identify any additional articles that would be relevant to this study. The articles retrieved from the database search and reference list (531) were transported to EndNote version 20, and duplicates (19) were identified and removed to produce 512 titles. Following the eventual title, abstracts, and full-text screening, the total number of appropriate articles for this study was 13.

### Selection criteria

Only studies that identified the factors associated with home management hypertension, regardless of the study setting, were included. The studies excluded were protocols, systematic studies (hospital service factors) that hinder diagnosis and self-monitoring of hypertension, and the factors related to the service provider (e.g., nurse, doctor), like shortage of items.

### Quality appraisal

The two authors independently used the Mixed Methods Appraisal Tool (MMAT) version 2018 to assess each study for quality [24,25]. The assessment results using this tool were compared between the two researchers for similarities. Where differences were found between the results from the authors, it was discussed until a consensus was achieved. The MMAT is a quality assessment tool that appraises the methodological quality of qualitative, quantitative, and mixed methods studies. The quantitative section appropriate for appraising the articles selected for this review assessed the aim of the study, study design, methodology, recruitment of study participants, and data collection methods, including analysis, presentation of results, and discussion, as well as the conclusions. Based on the assessment, the studies can be individually rated as high, moderate, or low quality. However, the subsequent views of Hong et al. 2018 emphasized that the researcher does not need to assess the overall quality of studies using this categorization [25]. In their paper, the authors strongly recommended that a detailed presentation of the appraised findings should be done. The components of the screening questions from the MMAT include whether there were (a) the presence of clear research questions and (b) collected data addressing the research question. Based on the consensus gained during the appraisal, all the articles received an affirmative response to the two screening questions above.

The appraisal sections that were relevant for this study were the descriptive quantitative section that assessed the following criteria: (1) relevance of sampling strategy, (2) representativeness of sample to the target population, (3) appropriateness of measurements, (4) risk of nonresponse bias, and (5) appropriateness of statistical analysis. All the studies (n = 11) met the criteria of the appropriateness of the sampling strategy to answer the research question except two studies [26,27]. One study [28] assessed methodological quality using the qualitative section. The results showed an affirmation of all the qualitative appraisal questions. Another study [29] was assessed under the mixed methods section and was also affirmative to all the questions.

### Data extraction and analysis

First, the two authors developed, discussed, and accepted a matrix to ensure comprehensiveness in the extracted data. The key parameters extracted from each study were the year, population and sample, study settings, outcome variable, main determinants of home care and self-care, and key findings. Second, the information of each study was transformed into narrative statements to enable the use of the thematic synthesis approach for data analysis [30,31]. Third, in this approach, codes were generated from the narratives formed; the codes were then linked to form subthemes, while similar themes were coalesced to form the main themes. The main themes incorporated in informing the report of this study were (1) the prevalence of control of BP, (2) sociodemographic factors, (3) treatment-related factors, (4) knowledge of management, and (5) knowledge of prevention of risk factors of hypertension.

## Results

**Figure 1:**
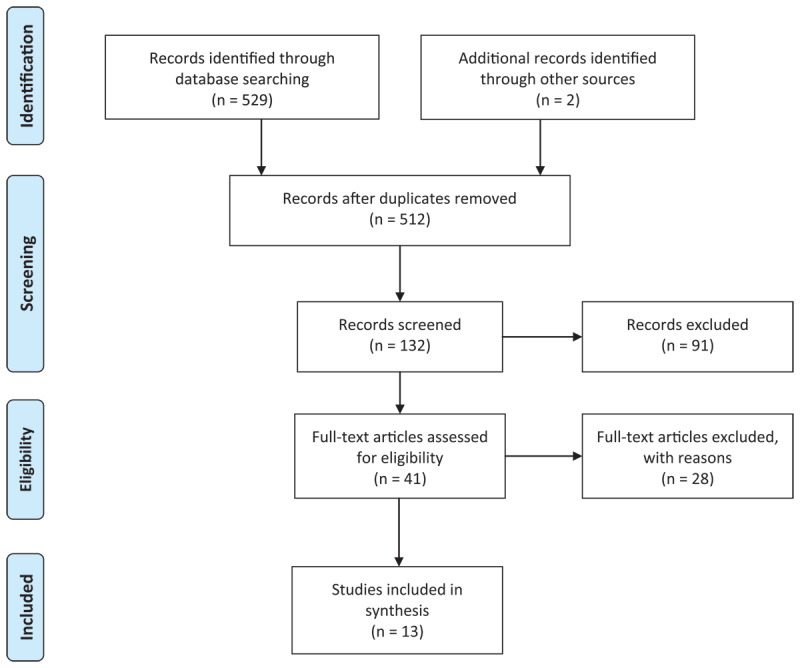
PRISMA flow diagram for selection of studies.

Through the advanced search of electronic databases using predetermined keywords (529) and a reference list of identified studies [2], about 531 studies were identified, and 19 were identified as duplicates in the endnotes. The duplicates were removed, and all 512 titles were screened through title and abstract. Based on a predetermined inclusion criterion, 41 studies were full articles, and 13 were identified as appropriate for this study. The process of article selection is shown in Figure 1.

### Study characteristics

The studies were conducted in various countries, including Tanzania [27], Ethiopia [32,33,34,35,36], Ghana [37], Sierra Leone [29], Cameroon [38], Eritrea [28], South Africa [26,39], and Kenya [40]. The various studies used diverse study designs. The characteristics of the studies used are shown in Table 1. The designs that were adopted in identifying the factors associated with home-based self-care management of hypertension in sub-Saharan Africa were cross-sectional [26,27,34,37,38], hospital-based cross-sectional [33,35,36], nationally representative cross-sectional data [39,40], retrospective cohort study [32], retrospective chart review [29], and qualitative study designs [28].

**Table 1:** Distribution of study characteristics.


AUTHOR	MAIN GOAL	SETTING	DESIGN	SAMPLE	DATA ANALYSIS

Maginga et al., 2015	Determine factors associated with BP control among adults attending a hypertension clinic	Bugando Medical Centre, Tanzania	Cross-sectional study	300 hypertension patients, selected consecutively	Fisher’s exact, Wilcoxon rank-sum test, univariable and multivariable logistic regression

Berhe et al., 2017	Examined determinants of achieving BP control and treatment intensification in patients with uncontrolled BP	Six public hospitals, Ethiopia	A retrospective cohort study	897 adult ambulatory hypertension patients	Descriptive statistics and multivariable logistic regression

Labata et al., 2019	Assessed predictors of self-care practices among hypertensive patients	Jimma University Specialized Hospital, Ethiopia	Hospital-based cross-sectional study	341 adult hypertensive patients	Descriptive statistics and multivariate logistics regression

Niriayo et al., 2019	Assessed the rate of adherence to self-care behaviors and associated factors among hypertensive patients	Ayder Comprehensive Specialized Hospital, Ethiopia	Cross-sectional study	276 ambulatory hypertensive patients	Univariable and binary logistic regression

Berhe et al., 2020	Assessed the prevalence and factors associated with uncontrolled hypertension among adults	Mekelle public hospitals, Tigray, Ethiopia	Hospital-based cross-sectional study	396 hypertensive patients, systematic random sampling	Bivariable and multivariable logistic regression

Gebremichael et al., 2019	Assessed self-care practices and associated factors among hypertensive patients	Ayder Comprehensive Specialized Hospital, Ethiopia	Hospital-based cross-sectional study	320 hypertension patients, simple random sampling	Descriptive statistics, logistics, and multivariate regression

Okai et al., 2020	Assessed the patient-level factors that influence hypertension control	Two hospitals in Accra, Ghana	Cross-sectional study	360 hypertensive patients	Chi-square tests and logistic regression

Herskind et al., 2019	Assessed an initiative conducted by two health clinics to begin treatment of hypertension among patients	Two clinics, Sierra Leone	Retrospective chart review and survey	487 records of patients and 68 hypertension patients’ convenience sample	Descriptive statistics

Adidja et al., 2018	Determine the rate and factors associated with nonadherence to antihypertensive pharmacotherapy, the association between nonadherence and BP control	Buea Health District, Cameroon	Community-based cross-sectional study	183 adults, stratified cluster sampling	Descriptive, chi-square, Fisher’s exact test, t-test, multivariable logistic regression

Gebrezgi et al., 2017	Identified barriers and facilitated hypertension management from the perspective of the patients	Asmara, Eritrea	Qualitative study	48 individual in-depth interviews and 2 FGD	Thematic analysis

Ware et al., 2019	Investigated traditional risk factors alongside other health and sociodemographic indicators to determine predictors of hypertension prevalence and management	South Africa	Cross-sectional of a nationally representative cohort	WHO-SAGE South Africa Wave 1 recruited 4,223 respondents from selected probability sampled	Chi-square, Mann-Whitney U test, t-tests, logistic regression

Mohamed et al., 2018	Estimated the prevalence of hypertension, awareness, treatment, and control	Kenya	A national cross-sectional household survey study	4,485 data from the 2015 Kenya STEPs survey, randomly selected	Descriptive statistics, multiple logistic regression, bivariate logistic regression

Adeniyi et al., 2016	Examined the sociodemographic and clinical determinants of uncontrolled hypertension among individuals living with T2DM in the rural communities	Mthatha, South Africa.	Cross-sectional study	265 individuals living with T2DM and hypertension	Univariate and multivariate logistic regression


**Legends:** BP = blood pressure; FGD = focus group discussion; T2DM = type 2 diabetes mellitus.

## Thematic Data Analysis

The synthesis showed that five key themes were associated with home-based self-care and BP control among patients. These key terms that emerged were (1) the prevalence of control of BP, (2) sociodemographic factors, (3) treatment-related factors, (4) knowledge of management, and (5) knowledge of prevention of risk factors of hypertension. The distribution of key findings is presented in Table 2.

**Table 2:** Distribution of key findings.


AUTHOR	OUTCOME AND MEASUREMENT	KEY DETERMINANTS OF HYPERTENSION CONTROL	KEY FINDINGS

Maginga et al., 2015	Medication adherence: MMAS-4 Knowledge: Self-developed pretested questionnaire	Good knowledge (OR = 2.50, 95% CI, 1.00–6.10, p = 0.047) Attitudes (OR = 2.70, 95% CI, 1.00–7.10, p = 0.004) Practices (OR = 5.40, 95% CI, 2.30–13.0, p < 0.001)	Patients (47.7%) had controlled hypertension. Obesity and higher medication costs were associated with decreased control. There was high adherence (56.0%) to medication. Participants had moderate scores for knowledge (41.0%), attitudes (45.3%), and practices (49.3%).

Berhe et al., 2017	Medication adherence: MMAS Other determinants: Self-developed questionnaire	Treatment at general hospitals (OR = 1.89, 95% CI 1.26–2.83) Previously uncontrolled BP (OR = 0.30, 95% CI 0.21–0.43) Treatment regimens with diuretics (OR = 0.68, 95% CI 0.50–0.94) Age (OR = 0.99, 95% CI = 0.98–1.00)	BP was controlled in 37.0%, and treatment was intensified for 23.0% of patients with uncontrolled BP. The antihypertensive medication adherence rate (MMAS ≥ 7) was 40.0% and 57.0% for the lower cutoff (MMAS ≥ 6).

Labata et al., 2019	Hypertension self-care practices: Adapted H-SCALE questionnaire	Normal weight (AOR = 1.82, 95% CI = 1.07–3.09) a predictor of medication usage Good self-efficacy (AOR = 2.58, 95% CI 1.47–0.52) a predictor of a low-salt diet Female predictor physical activity (AOR = 0.51, 95% CI 0.30–0.88) and nonsmoking (AOR = 3.62, 95% CI 1.21–10.85)	61.9%, 30.5%, 44.9%, 88.3%, 93.5%, and 56.9% were adherent to medication, low-salt diet, physical activity, alcohol abstinence, nonsmoking, and weight management, respectively. Adequate knowledge of hypertension was 2.58 times more likely, and females were less likely to adhere to physical activity.

Niriayo et al., 2019	Self-care behaviors: H-SCALE Beliefs about medication: Belief about medicine questionnaire (BMQ)	Rural resident (AOR = 0.45, 95% CI: 0.21–0.97) Comorbidity (AOR = 0.16, 95% CI 0.08–0.31) Negative medication belief (AOR = 0.25, 95% CI 0.14–0.46)	Antihypertensive medications adherent (48.2%) and recommended physical activity (44.9%) Female (AOR = 1.97, 95% CI 1.03–3.75) and lack of knowledge on self-care (AOR = 0.07, 95% CI 0.03–0.16) were associated with alcohol abstinence and a low-salt diet.

Berhe et al., 2020	Adherence to self-care activities: H-SCALE	Age ≥ 50 years (AOR = 2.33, 95% CI 1.25, 4.35) Nonadherence to antihypertensive medication (AOR = 1.82, 95% CI 1.08–3.04) Nonadherence to physical exercise (AOR = 1.79, 95% CI 1.13–2.83) Nonadherence to low-salt diet (AOR = 1.98, 95% CI 1.18–3.31) Nonadherence to weight management (AOR = 2.06, 95% CI 1.31–3.23)	Prevalence of uncontrolled hypertension was found to be 48.6%. 26.1%, 59.1%, 73.9%, and 38.6% of hypertensive patients were nonadherent to medication, physical exercise, low-salt diet, and weight management, respectively.

Gebremichael et al., 2019	Self-care practice: H-SCALE Knowledge: Hypertension evaluation of lifestyle and management (HELM) scale	Sex (AOR = 2.25, 95% CI 1.09–4.65) Age (AOR = 3.26, 95% CI 1.03–10.35) Educational status (AOR = 4.20, 95% CI 1.30–13.55) Disease duration (AOR = 3.12, 95% CI 1.20–8.10) BP status (AOR = 2.72, 95% CI 1.25–5.92) Knowledge (AOR = 6.19, 95% CI 2.90–13.21)	Good self-care practice was only found among 20.3% of patients. Adherence to not smoking, antihypertensive medication, alcohol abstinence, dietary management, physical exercise, and weight management was found to be 99.1%, 74.1%, 67.2%, 63.1%, 49.4%, and 40.6%, respectively.

Okai et al., 2020	Blood pressure control: Pretested self-developed questionnaire with expert opinion	Sex (AOR = 3.53, 95% CI 1.73–7.25) Educational at junior high school (AOR = 3.52, 95% CI 1.72–7.22) Senior and junior high school (AOR = 2.64, 95% CI 1.40–6.66 and AOR = 3.06, 95% CI 1.03–6.67) Comorbidity (AOR = 2.41, 95% CI 1.32– 4.42) Increased pill burden (AOR = 0.27, 95% CI 0.10–0.73) Length of diagnosis of 2–5 years (AOR = 0.32, 95% CI 0.18–0.57)	No comorbidities (18.0%) had achieved hypertension control. Dyslipidemia (8.9%) had controlled hypertension (p < 0.006). Taking a higher number of antihypertensive pills per day was also associated with a reduced likelihood of attaining hypertension control. Most patients reported forgetfulness, side effects of medication, and high pill burden as reasons for missing their medications.

Herskind et al., 2019	Medication adherence: Medication possession ratio	Patients were most likely to cite transportation (81.0%), financial burden (69.0%), and schedule conflicts with work or other prior commitments (25.0%) as barriers to care.	Forgetfulness (12.0%) and lack of symptoms (9.0%) were challenges that patients reported facing in attending follow-up appointments. Home visits (13.0%), outreach (13.0%), and phone or mobile reminders (12.0%) were strategies to improve adherence.

Adidja et al., 2018	Medication adherence: MMAS	Forgetfulness (AOR = 7.90, 95% CI 3.00–20.80) Multiple daily doses (AOR = 2.50, 95% CI 1.20–5.60) Financial constraints (AOR = 2.80, 95% CI 1.10–6.90) Adverse drug effects (AOR = 7.60, 95% CI 1.70–33.0)	Participants (67.7%) were nonadherent to medications. BP was controlled in only 21.3% of participants and was better in those who were adherent to medication (47.5%, p < 0.010).

Gebrezgi et al., 2017	Facilitators and barriers to self-care: Self-developed interview guide and focus group discussion guide		Individual factors: economic barriers, stress, nonadherence to medications due to the use of traditional remedies, and difficulties and misconceptions about following physical activity guidelines influenced self-care. Individual knowledge, family, and government support were important factors to the patient’s success in the personal hypertension management.

Ware et al., 2019	Predictors of hypertension prevalence and management: World Health Survey (WHS, 2002–2004; 70 countries)	Waist-to height ratio > 0.5 and diabetes comorbidity were the most significant predictors of hypertension presence, awareness, and treatment. Women and individuals reporting lower salt use were more likely to be aware of and treated for hypertension.	Older age, larger waist-to-height ratio, lower levels of education, and diabetes comorbidity were also predictive of individuals with hypertension being aware of their status. Older age, female sex, larger waist-to-height ratio, diabetes comorbidity, lower levels of education, and not adding salt to food at the table were predictive of current antihypertensive medication use.

Mohamed et al., 2018	World Health Organization’s STEPs survey methodology tool	Among those aware, only 26.9% were on treatment, and 51.7% among those on treatment had achieved blood pressure control. Factors associated with hypertension were older age, higher BMI, and harmful use of alcohol.	The overall age-standardized prevalence for hypertension was 24.5%. Only 15.6% were aware of their elevated blood pressure. Factors associated with awareness were older age (p = 0.013) and being male (p < 0.001).

Adeniyi et al., 2016	Uncontrolled hypertension: Self-developed questionnaire	Unemployed status (p < 0.001) Excessive alcohol intake (p = 0.007) Consumption of a Western-type diet (p < 0.001)	Independent determinants of uncontrolled hypertension were unemployment, current excessive drinker of alcohol and adherence to Western-type diet.


**Legends:** AOR = adjusted odds rations; BMI = body mass index; BMQ = belief about medicine questionnaire; CI = confidence interval; HELM = hypertension evaluation of lifestyle and management; MMAS = Morisky Medication Adherence Scale; WHS = World Health Survey.

### Prevalence of control of BP

Two studies specifically assessed the prevalence of hypertension control during their respective studies. It was shown that hypertension control prevalence during the study periods was 47.7% [27] and 37.0% [32].

### Sociodemographic factors associated with BP control

Several sociodemographic variables were assessed to determine the factors that influence hypertension control. The key subthemes assessed included gender (sex), age, and socioeconomic status.

#### Gender as a predictor of BP

The gender of hypertension patients was identified as an important factor associated with the ability to control BP [34,36]. Some of the studies showed that females (AOR = 2.25, 95% CI 1.09–4.65, p = 0.028) were found 2.25 times [36], 3.55 times (AOR = 3.55, 95% CI 1.72–7.22) more likely to have good self-care practice than males [37]. Also, men were identified to have reduced awareness of BP status [26,39,40]. Men had significantly reduced odds of being aware of their hypertensive status (AOR = 0.35, 95% CI 0.22–0.56) compared to women [40]. Another important predictor was that females had an increased tendency to be adherent to antihypertensive medication use [26,27,34,39]. Females had a higher tendency of BP control behavior, such as weight management (AOR = 0.46, 95% CI 0.23–0.92) and physical activity (AOR = 0.22, 95% CI 0.12–0.40) than males [34]. Females were also identified to have higher medication adherence and BP control [38]. However, some studies did not identify any significant difference in BP control across genders [26,27,33]. Control of BP was not significantly different between men and women [27]. Female respondents were less likely to adhere to physical activity (AOR = 0.51, 95% CI 0.30–0.88) and nonsmoking (AOR = 3.62, 95% CI 1.21–10.85) behavior [33].

#### Age as a predictor of BP control

The age of hypertension patients was identified as an important predictor of the ability to control BP [32,34,35,36,38,39]. Uncontrolled hypertension was 2.3 times (AOR = 0.19, 95% CI 0.06–0.61) higher among patients above 50 years compared to those above 60 [34]. Also, patients above 50 years were less adherent to weight management than younger individuals (18–35 years) [34]. Self-care practices of participants also improved with advancing age [36]. Patients under 65 years were 3.26 times (AOR = 3.26, 95% CI 1.03–10.35, p = 0.044) more likely to have good self-care practice than patients above 65 years [36]. Also, other studies showed that increasing age was associated with the tendency to be medication adherent and consequently maintain BP control [34,36].

#### The socioeconomic situation as a predictor of BP control

The socioeconomic status of hypertension patients was an important factor associated with BP control [27,34]. The major economic challenge associated with controlling BP was the medication cost and treatment [27]. The higher medication cost was associated with decreased odds of BP control at the study visit [27]. Odds of control (OR = 0.80, 95% CI 0.70–0.95, p = 0.010) decreased by 20% for every 10,000 Tanzanian shillings (TZS) spent on medication [27]. The place of residence influences the ability of respondents to adhere to medications [34]. Rural residents were less adherent to their medication (AOR = 0.45, 95% CI 0.21–0.97) than urban dwellers [34].

Other factors that predicted BP control and the self-care ability of respondents were the educational levels, as those who had at least a college level of education were found to be 4.205 times (AOR = 4.20, 95% CI 1.30–13.55, p = 0.016) more likely to have good self-care practice than those who were unable to read and write [36]. Participants with greater social support were 2.81 times (AOR = 2.81, 95% CI 1.20–6.53) more likely to adhere to a low-salt diet than their counterparts [33]. Other important socioeconomic factors that predicted control of hypertension were forgetfulness (AOR = 7.90, 95% CI 3.00–20.80, p < 0.001) and lack of finances (AOR = 2.80, 95% CI 1.10–6.90, p = 0.024) [34].

### Treatment-related factors

The treatment-related factors associated with home-based and self-care management of hypertension had three themes: (1) duration of treatment of hypertension, (2) pill-related factors, and (3) medication adherence.

#### Duration of treatment

Several important key factors influence the ability to continue treatment and ensure BP control [32,36,37]. Some of these factors included the duration of disease and treatment, significantly influencing the ability to initiate and maintain self-care [36,38] and control BP [32,38]. Less than four years of disease duration was 3.12 times (AOR = 3.12, 95% CI 1.20–8.10, p = 0.019) more likely to practice good self-care than those with less than < 2 years of disease duration [36].

#### Medication-related factors

The number of medications the hypertension patient took also influenced their ability to control and maintain self-care [26,37]. When hypertension patients took three to four antihypertensive pills daily, the odds of having a controlled BP were reduced by 68.0% (AOR = 0.32, 95% CI 0.18–0.57) compared to those who took one to two pills [37]. When patients take multiple antihypertensive agents, it improves their tendency to have controlled BP [26]. Also, multiple daily doses (AOR = 2.50, 95% CI 1.20–5.60, p = 0.020) and drug side effects (AOR = 7.00, 95% CI 1.70–33.6, p = 0.007) were independent predictors of nonadherence after controlling for potential confounders in multivariate analysis [38]. Other pill-related factors that influence the control of BP were follow-up at general hospitals (OR = 1.89, 95% CI 1.26–2.83), inadequately controlled BP at prior visits (OR = 0.30, 95% CI 0.21–0.43), longer treatment duration per year (OR = 1.04, 95% CI 1.01–1.06), and prescribed diuretics (OR = 0.68, 95% CI 0.50–0.94) [32].

#### Medication adherence to BP control

The level of medication adherence was determined to be high [27,32,34] and was measured using the Morisky Medication Adherence Scale [MMAS] [27,32]. Most patients (56.0%) had high adherence to medication on the MMAS-4 [27]. The antihypertensive medication adherence rate (MMAS ≥ 7) was 40.0% and 57.0% for the lower cutoff (MMAS ≥ 6) [32]. Patients with high medication adherence to the MMAS-4 had increased odds (OR = 18.8, 95% CI 7.80–45.40, p < 0.001) of control relative to those with low adherence at the study visit [27]. Patients with a negative medication belief were less likely to adhere to their medication (AOR = 0.25, 95% CI 0.14–0.46) than those with a positive medication belief [34]. Adult participants were adherent to the prescribed antihypertensive medications (48.2%) and the recommended level of physical activity (44.9%) [34]. Patients who were nonadherent to prescribed antihypertensive drugs were two times (AOR = 1.82, 95% CI 1.08–3.04) more likely to have uncontrolled hypertension than those who were adherent [35]. Participants with poor self-efficacy (AOR = 0.40, 95% CI 0.22–0.73) were less likely to adhere to medication usage than participants with good self-efficacy [33]. Regarding specific practices, rarely or never taking medications as prescribed (OR = 0.10, 95% CI 0.50–0.20, p < 0.001) was associated with decreased hypertension [HTN] control [27].

### Knowledge as a predictor of BP control

An important determinant of BP control was the level of knowledge of hypertension and control measures [27,34,36]. This was because moderate (OR = 1.80, 95% CI 1.01–3.20, p = 0.046) and good knowledge (OR = 2.10, 95% CI 1.00–4.50, p = 0.049) of HTN had increased odds of control [27]. Also, good knowledge was found 6.19 times (AOR = 6.19, 95% CI 2.90–13.21, p < 0.001) more positively associated with good self-care practice than poor knowledge [36]. Hypertension patients who knew the negative effects of salt (94.9%), alcohol (81.5%), and smoking (87.7%) and the positive effect of physical exercise (54.7%) had positive actions toward hypertension control [34]. Hypertension patients who were not knowledgeable about SCBs were less adherent to weight management (AOR = 0.13, 95% CI 0.03–0.57) and alcohol abstinence (AOR = 0.07, 95% CI 0.03–0.16) compared to those who were knowledgeable [34]. Overall, 82.2% of the participants were knowledgeable about the impact of the Self-care behavior [SCB] on hypertension control [34]. Those with moderate (OR = 2.80, 95% CI 1.20–6.40, p = 0.020) and good (OR = 3.00, 95% CI 1.30–7.00, p = 0.010) attitudes had increased odds of hypertension control [27]. Hypertension patients having adequate knowledge of hypertension were 2.58 times (AOR = 2.58, 95% CI 1.12–5.94) more likely to adhere to practicing physical activity [33].

### Knowledge of prevention of risk factors of hypertension

Several risk factors of hypertension were identified to influence the tendency to have good BP control. Some of these key risk factors include salt intake, practices, weight control, avoiding smoking and alcohol, and the presence of other comorbidities.

#### The level of salt in the diet was associated with BP control

The level of salt intake was an important predictor of hypertension control among patients, as nonadherents to a low-salt diet were two times (AOR = 1.98, 95% CI 1.18–3.31) more likely to develop uncontrolled hypertension [35]. Also, when individuals’, especially women’s, knowledge of hypertension was identified to be low, their chances of having a high-salt diet increased [39]. Another important factor was adding salt to meals (OR = 0.40, 95% CI, 0.20–0.60, p = 0.001) as an important factor for BP control [27].

#### Behavioral measures as a predictor of BP control

The conscious behavioral measures instituted by individuals were an important predictor of BP control among hypertension patients [27,32,36]. Controlled BP was found 2.7 times (AOR = 2.72, 95% CI 1.25–5.92, p = 0.011) more associated with good self-care practice than uncontrolled BP [36]. Those who had moderate (OR = 4.80, 95% CI 2.40–9.40, p < 0.001) and good (OR = 11.00, 95% CI 5.00–24.20, p < 0.001) practices to prevent hypertension also had increased odds of controlled BP [27]. More severe hypertension stages, stage II hypertension (OR = 0.17, 95% CI 0.09–0.35) and stage I hypertension (OR = 0.34, 95% CI 0.17–0.67), were associated with more difficulty in achieving the target BP [32].

#### Weight as a predictor of BP control

Hypertension patients who were more conscious of controlling body weight had a higher chance of BP control [33,35]. The odds (AOR = 2.06, 95% CI 1.31–3.23) of uncontrolled hypertension were twice as high among those with nonadherent weight management [35]. Normal weight patients were 1.82 times (AOR = 1.82, 95% CI 1.07–3.09) more likely to adhere to medication usage practice than overweight respondents [33,35], while normal weight respondents were 2.22 times more likely (AOR = 2.21, 95% CI 1.21–4.04) to practice weight management [33]. Hypertension patients with good self-efficacy were 2.60 times more likely (AOR = 2.58, 95% CI 1.41–4.73) to maintain their weight than poor self-efficacy [33]. Also, rarely or never adhering to normal weight control advice (OR = 0.40, 95% CI 0.20–0.60, p < 0.001) was a predictor of poor hypertension control [27]. Patients who did not adhere to physical exercise were 1.8 times (AOR = 1.79, 95% CI 1.13–2.83) more likely to have uncontrolled hypertension compared to those who adhered to physical exercise [35].

#### Smoking and alcohol intake are predictors of high BP

Alcohol intake was identified as an important factor that influenced the likelihood of having hypertension control [27,33]. This was because drinking alcohol (OR = 0.30, 95% CI 0.10–0.70, p = 0.006) was a significant factor in BP control [27,33]. Females were more likely to adhere to alcohol abstinence (AOR = 1.97, 95% CI 1.03–3.75) and nonsmoking behavior (AOR = 6.33, 95% CI 1.80–22.31) than males [34].

#### Comorbidities as a predictor of BP control

The level and presence of comorbidities were identified to be associated with hypertension patients’ ability to have controlled BP [34,37,39]. Patients with comorbidities were also less adherent to their medication (AOR = 0.16, 95% CI 0.08–0.31) than those without comorbidities [34]. Also, a 69.0% (AOR = 0.31, 95% CI 0.11–0.89) reduction in the odds of having controlled hypertension was identified among patients who suffered from dyslipidemia as a comorbidity [37]. Elevated waist-to-height ratio and diabetes diagnosis were the most significant predictors of hypertension and being aware of hypertension status [39].

## Discussion

This review identified and integrated the factors associated with home-based self-care management of hypertension. In chronic diseases like hypertension, home-based management is cardinal in improving a patient’s outcome and the ability to avoid complications and limit the progress of the disease [41,42,43]. Hypertension patients must identify and institute home measures that help them improve their BP levels and avoid related complications. The studies on hypertension home-based management are largely cross-sectional and limited to a specific culture or geographical location. Therefore, we were motivated to identify the factors associated with home-based management of hypertension to identify and integrate measures to improve client outcomes. We identified the prevalence of BP control to range from 21.3% [38] to 47.7% [27] among hypertension patients.

This study highlighted the diverse components that must be considered when interventions are implemented for home-based self-care management of hypertension. Hypertension control through self-care at home was reported in similar systematic reviews to be influenced by diverse factors [41]. An important point of this home management involves measuring BP [42,43]. Self-care management of hypertension was instrumental when it was noted that intervention efficacy is most felt when the patient is used as the change agent [44]. Poor self-management and medication adherence were identified to negatively influence hypertension control among patients [9].

In this study, we identified multiple interactive factors that influence home-based self-care and management of BP. These factors include sociodemographic, treatment-related, and knowledge of hypertension management and risk factors. Divergent and multiple nature of the factors that are identified to influence hypertension control in Africa warrants commensurate measures to eliminate the associated repercussions of the disease. The culmination of these interventions must focus on reducing hypertension risk, ensuring medication adherence, and promoting appropriate lifestyle modifications.

The divergent factors of multilevel, multicomponent interventions will ensure and promote comprehensive solutions (Mills et al., 2018), especially in lower- and middle-income countries like those in sub-Saharan Africa. This strategy is imperative to limit the challenges imposed by high BP. The sociodemographic factors that influence hypertension control through the home-based self-care ability of patients were gender (female), age (older), place of residence (urban), educational level (educated), and socioeconomic status (high). These sociodemographic characteristics are predictive of hypertension as explanatory variables [16,17,40,45,46,47,48], yet they were identified to influence the ability of patients to control hypertension. While little can be done about sociodemographic factors as a risk to hypertension (nonmodifiable risk), identifying these factors gives a category of where the emphasis should be placed on preventing and controlling hypertension. Home management education must prioritize categories that have inadequate skills in home management. To promote home-based self-care management of hypertension in lower- and middle-income countries, intervention studies must focus on identifying the influence of each factor on the care of the patient and their ability to institute home-based management of hypertension.

Increasing knowledge on control measures, including limiting risk factors, and improving medication adherence through health education will be central in assisting patients in improving home-based care. In a related systematic review and meta-analysis, this study demonstrated that the number of pills and intake duration strongly influence adherence to medication at home, especially among patients with hypertension [49]. Patients who were noted to take antihypertensive medications over a long period with few medications were said to have higher adherence levels. These factors were important in other systematic reviews [49,50,51]. It is important that when patients are discharged from the hospital to home for self-care management, only relevant (few) medications with limited pill dosage requirements will promote a positive attitude toward care and ensure hypertension control. It was noted that coordinated interventions used in managing hypertension that limit pill number and frequency among patients and increase knowledge and lifestyle changes are useful [52,53].

Other important themes that emerged were the level of knowledge on hypertension and the presence of risk factors (modifiable risk factors) of hypertension. These modifiable risk factors are mainly centered on knowledge levels, smoking, alcohol, BMI, level of salt intake, and the presence of comorbidities. It was shown that even though this factor predicts the presence of hypertension, it also influences the level of treatment patients adopt during home management. The impact of these modifiable risk factors on the ability to control hypertension, especially in lower- and middle-income countries, was also documented in a previous study [54,55,56]. To improve knowledge and, at the same time, eliminate the risk associated with hypertension, interactive technological methods will increase the likelihood of behavior change, which is implicated in the inability to achieve successful home-based self-care management [12,52].

Research and policy making should streamline intervention studies that will improve home-based and self-care management of hypertension, especially in low-resource settings. In this review, we identified that important interventions that will improve self-care and home management of hypertension should focus on improving self-efficacy, increasing knowledge, and targeting specific attitudes of patients to improve adherence. Also, intervention research methods must focus on eliminating the risk factors of hypertension and segregating patients based on demographic categorization (like age, gender, socioeconomic status, and level of education) for implementation.

## Conclusion

This systematic review identified and synthesized the factors associated with home-based and self-care management of hypertension. These factors are complex and multi-sectoral to improve the lives of people with hypertension; multi-sectoral approaches are their force required. Hypertension self-care interventions in lower resource settings must incorporate individual, societal, and cultural perspectives in increasing knowledge and improving home-based hypertension management. In this review, it could be seen that the use of technology-based interventions for improving home-based self-care management of hypertension is limited. This warrants the use of technology-based intervention (including social media networks and phone-based text messaging) that could improve teaching, coaching, and monitoring and give feedback to patients, especially when they engage in home-based self-care management of hypertension. Also, well-designed clinical experimental studies use complex interventions to increase knowledge and self-efficacy and improve behaviors toward home management of hypertension.

## Data Accessibility Statement

All data from which the conclusions of this study were made are included in this manuscript, and no data is deposited in any public database.

## Additional File

The additional file for this article can be found as follows:

10.5334/gh.1190.s1 Supplementary File. Results of quality appraisal using the mixed methods appraisal tool.
