# Supplementary material for: Determinants of Self-Care and Home-Based Management of Hypertension: An Integrative Review
Source: Glob Heart. 2023 Mar 20;18(1):16. doi: 10.5334/gh.1190 (PMC10038107; doi:10.5334/gh.1190)
Supplement: Supplementary File. — Results of quality appraisal using the mixed methods appraisal tool. [file gh-18-1-1190-s1.pdf]

Results of quality appraisal using the mixed methods appraisal tool

QUANTITATIVE DESCRIPTIVE STUDY								
		Screening questions		Appraisal questions				
Citation	Specific study design	Are there clear research questions?	Do the collected data allow to address the research questions?	Is the sampling strategy relevant to address the research question?	Is the sample representative of the target population?	Are the measurements appropriate?	Is the risk of nonresponse bias low?	Is the statistical analysis appropriate to answer the research question?
Maginga et al., 2015	Cross sectional	Yes	Yes	No	Yes	Yes	Yes	Yes
Berhe et al., 2017	A retrospective cohort	Yes	Yes	Yes	Yes	Yes	Yes	Yes
Labata et al., 2019	Hospital-based cross-sectional	Yes	Yes	Yes	Yes	Yes	Yes	Yes
Niriayo et al., 2019	Cross sectional	Yes	Yes	Yes	Yes	Yes	Yes	Yes
Berhe et al., 2020	Hospital-based cross-sectional	Yes	Yes	Yes	Yes	Yes	Yes	Yes
Gebremichael et al., 2019	Hospital-based cross-sectional	Yes	Yes	Yes	Yes	Yes	Yes	Yes
Okai et al., 2020	Cross sectional	Yes	Yes	Yes	Yes	Yes	Yes	Yes
Adidja et al., 2018	Community-based cross-sectional	Yes	Yes	Yes	Yes	Yes	Yes	Yes
Ware et al., 2019	Cross-sectional within a cohort	Yes	Yes	Yes	Yes	Yes	Yes	Yes
Mohamed et al., 2018	Cross-sectional household survey	Yes	Yes	Yes	Yes	Yes	Yes	Yes

Adeniyi et al., 2016	Cross-sectional	Yes	Yes	No	Yes	Yes	Yes	Yes
**QUALITATIVE STUDY**								
	Specific design	Are there clear research questions?	Do the collected data allow to address the research questions?	Is the qualitative approach appropriate to answer the research question?	Are the qualitative data collection methods adequate to address the research question?	Are the findings adequately derived from the data?	Is the interpretation of results sufficiently substantiated by data?	Is there coherence between qualitative data sources, collection, analysis and interpretation ?
Gebrezgi et al., 2017	Qualitative	Yes	Yes	Yes	Yes	Yes	Yes	Yes
**MIXED METHODS STUDIES**								
citation	Specific design	Are there clear research questions?	Do the collected data allow to address the research questions?	Is there an adequate rationale for using a mixed methods design to address the research question?	Are the different components of the study effectively integrated to answer the research question?	Are the outputs of the integration of qualitative and quantitative components adequately interpreted?	Are divergences and inconsistencies between quantitative and qualitative results adequately addressed?	Do the different components of the study adhere to the quality criteria of each tradition of the methods involved?
Herskind et al., 2019	Retrospective chart review and survey: a mixed-method	Yes	Yes	Yes	Yes	Yes	Yes	Yes
